# Early in-hospital triglyceride clearance trajectories and time-weighted mean exposure in hypertriglyceridemia-associated acute pancreatitis: associations with disease severity and pancreatic necrosis

**DOI:** 10.3389/fmed.2026.1921788

**Published:** 2026-09-09

**Authors:** Jiali Cui, Jie Li, Zhining Sun, Jiajia Zhao, Huili Wu

**Affiliations:** Department of Gastroenterology, Zhengzhou Central Hospital Affiliated to Zhengzhou University, Zhengzhou, China

**Keywords:** hypertriglyceridemia-associated acute pancreatitis, pancreatic necrosis, severe acute pancreatitis, time-weighted mean triglyceride exposure, triglyceride trajectory

## Abstract

**Background:**

Hypertriglyceridemia-associated acute pancreatitis (HTG-AP) is a clinically important subtype of acute pancreatitis associated with adverse outcomes. Previous assessments have mainly relied on admission or peak triglyceride (TG) levels, which may not capture early in-hospital TG clearance dynamics or sustained exposure intensity. This study investigated the associations of TG clearance trajectories and time-weighted mean triglyceride exposure (TWM-TG) with severe acute pancreatitis (SAP) and pancreatic necrosis in HTG-AP.

**Methods:**

This retrospective cohort study included 526 patients with HTG-AP. Serial TG measurements obtained within 7 days after admission were analyzed. A latent class mixed model (LCMM) was used to identify TG clearance trajectories. The area under the TG exposure curve was calculated using the trapezoidal rule and standardized by the interval between the first and last TG measurements to derive TWM-TG. Missing data were handled using multiple imputation by chained equations. Covariates were selected using the Boruta algorithm, followed by LASSO regression with 10-fold cross-validation and variance inflation factor assessment. Firth logistic regression was performed in five imputed datasets, and estimates were pooled using Rubin's rules.

**Results:**

Two TG trajectories were identified: a rapid-decline group (n=430) and a slow-decline group (n=96). The proportion of patients with SAP was higher in the slow-decline group than in the rapid-decline group (46.9% vs 11.9%). After multivariable adjustment, a slow-decline TG trajectory (OR=6.44, 95% CI: 3.55–11.69, *P* < 0.001) and TWM-TG (OR=1.66, 95% CI: 1.33–2.08, *P* < 0.001) were associated with higher odds of SAP, whereas maximum TG was not significantly associated with SAP. For pancreatic necrosis, only TWM-TG remained significantly associated (OR=1.27, 95% CI: 1.05–1.54, *P* = 0.016).

**Conclusion:**

TG clearance trajectories and TWM-TG were associated with disease severity in HTG-AP and may provide supplementary information beyond static TG measurements. TWM-TG was also associated with pancreatic necrosis, suggesting relevance to local pancreatic injury. Further multicenter validation is warranted to confirm the generalizability and clinical relevance of these findings.

## 1 Introduction

Driven by the global rise in obesity, diabetes, and metabolic syndrome, hypertriglyceridemia (HTG) has emerged as a clinically distinct and increasingly prevalent etiology of acute pancreatitis (AP) (1, 2). This trend is particularly evident in China, where recent epidemiological studies indicate that HTG has surpassed alcohol to become the second leading cause of AP (3, 4). Although clinical outcomes vary, accumulating evidence suggests that, compared with other etiologies, hypertriglyceridemia-associated acute pancreatitis (HTG-AP) frequently presents with a more aggressive early clinical course, including a greater frequency of local complications and intensive care admission (5). However, because both the systemic inflammatory response and the underlying lipid profile may fluctuate rapidly during the early phase of the disease, relying solely on static baseline evaluations to characterize early disease severity and local pancreatic injury remains clinically challenging (6).

Despite this recognized limitation, routine assessment of HTG-AP severity still predominantly depends on static scoring systems and isolated biochemical parameters, with baseline or peak triglyceride (TG) levels commonly used to estimate the initial lipid burden (3, 7, 8). However, these static snapshots may not adequately reflect the ongoing metabolic changes during the early in-hospital disease course. Following early clinical interventions—ranging from fasting and fluid resuscitation to insulin therapy, lipid-lowering treatment, or plasma exchange—patients may exhibit highly variable TG clearance patterns (9–11). While some patients achieve rapid lipid reduction, others experience delayed or slower clearance. This temporal heterogeneity may represent clinically relevant disease-course information, providing insight into sustained TG exposure and metabolic burden that baseline or peak values alone may not fully capture.

Alongside the dynamic pattern of TG clearance, the average intensity of early in-hospital TG exposure may also have important biological relevance (12). Sustained severe hypertriglyceridemia may be involved in local and systemic injury through free fatty acid–mediated lipotoxicity, pancreatic microcirculatory impairment, and amplification of systemic inflammatory responses (13, 14). In this context, time-weighted mean TG exposure (TWM-TG), a metric reflecting the average exposure intensity over a defined observation period, may complement isolated baseline or peak values in characterizing early lipid metabolic burden (15). Despite this biologically plausible rationale, systematic studies evaluating the clinical relevance of early in-hospital TG clearance patterns and TWM-TG in patients with HTG-AP remain limited.

To address this gap, the present retrospective cohort study of 526 patients with HTG-AP systematically analyzed dynamic changes in TG levels within the first 7 days after admission. We used a latent class mixed model to identify major patterns of early in-hospital TG clearance and quantified the average lipid exposure burden by calculating TWM-TG after standardizing the area under the TG exposure curve by observation time. We then evaluated the associations of TG clearance trajectories, TWM-TG, and maximum TG with severe acute pancreatitis (SAP) and pancreatic necrosis. Furthermore, restricted cubic spline analysis was performed to explore potential nonlinear associations, and subgroup analyses were conducted to examine whether the association between TG trajectories and SAP was consistent across subgroups with different baseline clinical characteristics.

## 2 Methods

### 2.1 Study design and study population

This single-center retrospective cohort study included consecutive patients with HTG-AP who were admitted to Zhengzhou Central Hospital Affiliated to Zhengzhou University between January 2018 and April 2026. A total of 840 consecutive patients with HTG-AP were screened during the study period. After screening according to predefined inclusion and exclusion criteria, 526 patients were ultimately included in the analyses of TG trajectories and TWM-TG. AP was diagnosed according to the 2012 revised Atlanta classification, requiring the presence of at least two of the following three criteria: (1) typical acute upper abdominal pain; (2) serum amylase and/or lipase levels greater than three times the upper limit of normal; and (3) imaging findings suggestive of acute pancreatitis (16).

HTG-AP was diagnosed when all of the following criteria were met: (1) fulfillment of the diagnostic criteria for AP; (2) hypertriglyceridemia was identified as the etiology, defined as a serum TG concentration ≥1000 mg/dL (11.3 mmol/L), or a serum TG concentration of 500–1000 mg/dL (5.65–11.3 mmol/L) accompanied by chylous serum; and (3) exclusion of AP caused by biliary, alcohol-related, drug-induced, traumatic, tumor-related, autoimmune, or other clearly identifiable etiologies (16).

The inclusion criteria were as follows: (1) fulfillment of the 2012 revised Atlanta diagnostic criteria for AP; (2) fulfillment of the above diagnostic criteria for HTG-AP; and (3) availability of serial TG measurements during the first 7 days after admission suitable for trajectory modeling. The exclusion criteria were as follows: (1) age <18 years; (2) hospital stay <7 days; (3) fewer than three TG measurements during the first 7 days after admission; (4) concurrent end-stage liver failure or end-stage renal failure; and (5) pregnancy. The requirement for a hospital stay of at least 7 days and at least three valid TG measurements during the first 7 days was used to ensure sufficient longitudinal TG data for trajectory modeling during the first 7 days and estimation of TWM-TG. Baseline clinical characteristics, admission or early in-hospital laboratory findings, available treatment-related information, dynamic TG measurements during the first week after admission, and outcome data were collected for all included patients. The study protocol was approved by the Ethics Committee of Zhengzhou Central Hospital Affiliated to Zhengzhou University (approval number: ZXYY2026060-1). Because this was a retrospective study, the requirement for informed consent was waived by the Ethics Committee.

### 2.2 Data collection and variable definitions

Demographic characteristics, admission clinical data, medical history, laboratory parameters, and outcome data were collected. Baseline variables included age, sex, height, weight, time from disease onset to admission, underlying diseases, complete blood count, inflammatory markers, electrolytes, liver and renal function parameters, blood glucose and lipid profiles, coagulation function, amylase, lipase, and related indicators. Laboratory and clinical variables used as baseline covariates were obtained from admission records or the earliest available records after admission. Body mass index (BMI) was calculated from body weight and height. Dynamic TG data were obtained from all valid TG measurement records during the first 7 days after admission. The primary outcome was severe acute pancreatitis (SAP), defined as persistent organ failure lasting >48 h. The secondary outcome was pancreatic necrosis, including isolated pancreatic parenchymal necrosis, peripancreatic fat necrosis, and combined pancreatic parenchymal and peripancreatic fat necrosis (16, 17). Pancreatic necrosis was identified based on contrast-enhanced computed tomography (CECT) findings documented in formal radiology reports and clinical records. For patients diagnosed with pancreatic necrosis, the timing of the imaging examination used to confirm necrosis was recorded as the number of days from admission to the corresponding CECT examination.

TWM-TG was calculated using the trapezoidal integration method (18). For each patient, all TG measurements obtained during the first 7 days after admission were ordered chronologically by measurement time. The TG exposure area between two adjacent measurements was defined as the mean of the two TG values multiplied by the corresponding time interval. The exposure areas across all adjacent time intervals were summed to obtain the TG exposure area during the actual observation period. This TG exposure area was then divided by the time interval between the first and last TG measurements to derive TWM-TG, reflecting the average intensity of TG exposure per unit time during the actual observation period. The formula was as follows: TWM-TG = {*Σ*[(TG_i_ + TG_(i_ _+_ _1)_)/2 × *Δ*t_i_]}/(t_last_−t_first_), where *Δ*t_i_ denotes the time interval between two adjacent TG measurements, and t_last_ and t_first_ denote the times of the last and first TG measurements, respectively.

### 2.3 Modeling of dynamic TG trajectories

A latent class mixed model (LCMM) was used to identify dynamic trajectories of TG levels during the first 7 days after admission (19). Given the markedly right-skewed distribution of TG values, TG was natural log-transformed before trajectory modeling, and log(TG) was used as the longitudinal outcome variable. Time was measured as the actual number of days from admission to each TG measurement, thereby retaining irregular measurement intervals from routine clinical practice rather than forcing TG values into fixed daily time points. A quadratic term for time was included to capture potential nonlinear changes in TG levels over time. Patient identification number was used as the individual identifier, and a random effect for time was specified.

Latent class models with 2–4 classes were fitted separately. Model selection was based on convergence status, the Bayesian information criterion (BIC), class size, and class-specific average posterior probability (20). A model was considered to have acceptable classification performance if it successfully converged, each class accounted for ≥5% of the sample, and the minimum class-specific average posterior probability was >0.70. Among models meeting these criteria, the final model was selected according to model fit, posterior classification quality, class size, and clinical interpretability. Posterior classification quality for the candidate models was further summarized using class-specific assigned posterior probabilities, posterior probability matrices, overall mean assigned posterior probability, and relative entropy. The resulting trajectory groups were interpreted as broad empirical patterns of early in-hospital TG clearance rather than definitive biological subgroups. In subsequent analyses, the rapid-decline TG trajectory group was used as the reference group.

### 2.4 Machine learning-based variable selection

Candidate clinical covariates were selected separately for severe acute pancreatitis and pancreatic necrosis using a structured variable-selection procedure. First, the Boruta random forest algorithm was applied to identify candidate baseline covariates associated with each outcome. Subsequently, variables confirmed by the Boruta algorithm were entered into LASSO regression models, and 10-fold cross-validation was used to determine the optimal penalty parameter. Variables with nonzero regression coefficients under the optimal penalty parameter were retained. To reduce the influence of multicollinearity, variance inflation factors (VIFs) were further calculated, and variables with VIF >5 were excluded. The retained variables were used to construct the multivariable adjustment models for the corresponding outcomes.

### 2.5 Statistical analysis

Continuous variables were expressed as medians with interquartile ranges according to their distributional characteristics, and between-group comparisons were performed using the Wilcoxon rank-sum test. Categorical variables were expressed as numbers and percentages, and between-group comparisons were performed using the *χ*^2^ test or Fisher's exact test. All statistical tests were two-sided, and *P* < 0.05 was considered statistically significant.

Variables with a missing rate >20% were excluded. Variables with a missing rate <20% were imputed using multiple imputation by chained equations (MICE), generating five imputed datasets. Key regression models were fitted separately in each imputed dataset, and effect estimates and standard errors were pooled according to Rubin's rules, from which 95% CIs and P values were derived.

Firth logistic regression was used to analyze the associations between TG-related indicators and study outcomes, thereby reducing small-sample bias and the influence of complete or quasi-complete separation on model estimation (21, 22). The primary outcome was severe acute pancreatitis, and the secondary outcome was pancreatic necrosis. The TG-related indicators of interest included TG trajectory, TWM-TG, and maximum TG. TWM-TG and maximum TG were standardized, and effect estimates were calculated per 1-standard-deviation increase. In the TG trajectory analysis, the rapid-decline TG trajectory group was used as the reference group for comparison with the slow-decline trajectory group. Unadjusted and multivariable-adjusted models were constructed separately; the adjusted models included covariates selected using the Boruta algorithm, LASSO regression, and variance inflation factor (VIF) assessment (23, 24). These covariates were included to account for measured differences in baseline and early in-hospital clinical status and to assess whether the associations of TG-related indicators with the study outcomes persisted after multivariable adjustment. Because some of these variables may themselves reflect early disease severity, we interpret the resulting estimates as adjusted associations rather than causal effects. Results are presented as odds ratios (ORs) with 95% confidence intervals (CIs).

Restricted cubic spline (RCS) analysis was performed to assess the potential nonlinear association between TWM-TG and the study outcomes. RCS knots were placed at the 5th, 35th, 65th, and 95th percentiles of the TWM-TG distribution, with the median TWM-TG value used as the reference (25). Wald tests were used to evaluate the overall and nonlinear associations. Potential clinical thresholds were further explored only when statistical evidence of nonlinearity was present.

As a supplementary analysis, exploratory receiver operating characteristic (ROC) curve analysis was performed to compare the discriminatory information provided by TG-related indicators and clinical covariate models with respect to severe acute pancreatitis (26). The variables and models assessed included baseline TG, maximum TG, TWM-TG, TG trajectory, the clinical covariate model alone, and models combining TG trajectory and/or TWM-TG with clinical covariates. For individual TG-related indicators, ROC curves were constructed using standardized baseline TG, maximum TG, TWM-TG, and the TG trajectory grouping variable. For multivariable models, individual model-derived fitted probabilities were calculated using Firth logistic regression and then used to construct ROC curves. AUCs were calculated separately in the five imputed datasets and summarized as the mean AUC, mean 95% CI, and AUC range. The DeLong test was performed in the first imputed dataset to compare, on an exploratory basis, the discriminatory ability of baseline TG with that of TWM-TG, maximum TG, and TG trajectory, and to compare the discriminatory ability of the clinical covariate model alone with that of models incorporating TWM-TG and/or TG trajectory (27).

To evaluate whether the association between TG trajectory and severe acute pancreatitis was consistent across patient subgroups, subgroup analyses were performed. Prespecified subgroups included age, sex, BMI, diabetes status, and fatty liver status. Age was stratified according to the median value in this cohort, and BMI was stratified using 28 kg/m^2^ as the cutoff. Effect modification across subgroups was assessed by including interaction terms between TG trajectory and subgroup variables in the models (28).

All statistical analyses were performed using R software version 4.6.0. The main R packages used included mice, lcmm, Boruta, glmnet, logistf, Hmisc, pROC, ggplot2, and openxlsx.

## 3 Results

### 3.1 Study population screening and identification of TG trajectories

A total of 840 consecutive patients with HTG-AP were screened during the study period. After applying the predefined inclusion and exclusion criteria, 314 patients were excluded, and 526 patients were ultimately included in the final analysis. Baseline characteristics and clinical outcomes are presented in Table 1. All patients had at least three valid TG measurements during the first 7 days after admission, which were used for dynamic TG trajectory modeling. The distribution of TG measurement time points used for trajectory modeling is summarized in Supplementary Table 1. The median number of TG measurement time points was 3 (IQR: 3–3) in the rapid-decline trajectory group and 3 (IQR: 3–4) in the slow-decline trajectory group. The first TG measurement was obtained at a median of 0.05 days after admission in both groups, and the median observation intervals were similar between the rapid-decline and slow-decline trajectory groups (5.43 and 5.54 days, respectively).

**Table 1:** Baseline characteristics and clinical outcomes of patients according to TG trajectory group.

Variable	Overall	Rapid.decline	Slow.decline	P.value	Missing
Age, years	37.00 [32.00-43.00]	37.00 [32.00-43.00]	35.00 [31.00-42.00]	0.151	1 (0.2%)
BMI, kg/m2	27.68 [25.31-30.42]	27.68 [25.25-30.42]	27.70 [25.71-30.12]	0.936	8 (1.5%)
Baseline TG, mmol/L	20.09 [12.56-31.96]	19.60 [12.73-30.80]	22.08 [11.76-35.99]	0.334	0 (0.0%)
Maximum TG, mmol/L	20.80 [13.48-32.21]	20.30 [13.52-31.23]	22.83 [13.41-38.14]	0.071	0 (0.0%)
TWM-TG	8.64 [6.26-12.06]	7.99 [5.97-10.80]	13.00 [9.90-19.60]	<0.001	1 (0.2%)
Onset-to-admission time, d	0.50 [0.25-1.00]	0.50 [0.25-1.00]	0.56 [0.28-1.00]	0.063	0 (0.0%)
WBC, ×10^9/L	12.50 [10.16-15.55]	12.69 [10.06-15.61]	12.15 [10.42-14.49]	0.401	0 (0.0%)
CRP, mg/L	13.29 [3.54-66.21]	11.82 [3.15-67.31]	20.17 [6.15-65.47]	0.046	0 (0.0%)
Amylase, U/L	184.00 [85.50-417.00]	188.00 [89.00-412.50]	178.00 [71.75-476.00]	0.636	3 (0.6%)
Lipase, U/L	204.00 [103.75-466.25]	204.50 [106.75-472.25]	198.50 [99.00-401.00]	0.680	6 (1.1%)
BUN, mmol/L	4.72 [3.75-5.87]	4.53 [3.70-5.70]	5.31 [4.27-6.55]	<0.001	0 (0.0%)
Serum creatinine, μmol/L	51.70 [38.10-66.58]	51.40 [38.10-65.00]	53.45 [36.00-69.50]	0.314	0 (0.0%)
Calcium, mmol/L	2.30 [2.18-2.41]	2.30 [2.18-2.41]	2.30 [2.16-2.40]	0.740	0 (0.0%)
Procalcitonin, ng/mL	0.07 [0.02-0.21]	0.06 [0.02-0.19]	0.08 [0.03-0.32]	0.068	101 (19.2%)
Hematocrit, %	44.40 [41.40-47.30]	44.40 [41.40-47.11]	44.50 [41.68-47.85]	0.216	0 (0.0%)
Hemoglobin, g/L	157.00 [145.00-168.00]	157.00 [144.00-168.00]	161.00 [149.75-172.50]	0.020	0 (0.0%)
Albumin, g/L	43.00 [40.00-46.00]	43.20 [40.20-46.00]	42.85 [38.50-46.32]	0.723	1 (0.2%)
LDH, U/L	237.50 [194.50-293.25]	237.00 [200.00-289.00]	248.00 [181.50-315.00]	0.658	30 (5.7%)
Blood purification	14 (2.7%)	12 (2.8%)	2 (2.1%)	1.000	0 (0.0%)
Lipid-lowering therapy				0.064	0 (0.0%)
None	56 (10.6%)	46 (10.7%)	10 (10.4%)		
Fibrate	372 (70.7%)	310 (72.1%)	62 (64.6%)		
Statin	29 (5.5%)	26 (6.0%)	3 (3.1%)		
Fibrate + statin	56 (10.6%)	38 (8.8%)	18 (18.8%)		
Evolocumab + fibrate	13 (2.5%)	10 (2.3%)	3 (3.1%)		
Female sex	114 (21.7%)	95 (22.1%)	19 (19.8%)	0.720	0 (0.0%)
Diabetes mellitus	280 (53.2%)	218 (50.7%)	62 (64.6%)	0.019	0 (0.0%)
Hypertension	97 (18.5%)	78 (18.2%)	19 (19.8%)	0.824	1 (0.2%)
Fatty liver	419 (79.7%)	340 (79.1%)	79 (82.3%)	0.569	0 (0.0%)
Severe acute pancreatitis	96 (18.3%)	51 (11.9%)	45 (46.9%)	<0.001	0 (0.0%)
Pancreatic necrosis	149 (28.3%)	112 (26.0%)	37 (38.5%)	0.020	0 (0.0%)

Continuous variables are presented as median [interquartile range (IQR)], and categorical variables are presented as number (percentage). Between-group comparisons were performed using the Wilcoxon rank-sum test, *χ*^2^ test, or Fisher's exact test, as appropriate.

TG, triglyceride; TWM-TG, time-weighted mean triglyceride exposure; WBC, white blood cell count; CRP, C-reactive protein; BUN, blood urea nitrogen; SAP, severe acute pancreatitis.

The LCMM model selection results are presented in Table 2. All candidate LCMMs with 2, 3, and 4 classes converged. Although the three-class model had the lowest AIC, the two-class model had the lowest BIC and was the only model meeting the predefined eligibility criteria, including successful convergence, minimum class proportion ≥5%, and minimum class-specific average posterior probability >0.70. It also showed the most favorable overall classification quality. In contrast, the three- and four-class models showed poorer classification quality, with lower minimum class-specific mean assigned posterior probabilities and lower relative entropy values. Therefore, the two-class model was selected as the final trajectory model. Detailed posterior classification quality indices are provided in Supplementary Table 2.

**Table 2:** Model Fit and classification criteria for dynamic TG trajectory models.

Model	No. of classes	Converged	AIC	BIC	Minimum class proportion	Minimum class-specific average posterior probability	Relative entropy	Eligible
ng2	2	Yes	3390.76	3441.94	0.183	0.784	0.533	Yes
ng3	3	Yes	3388.66	3461.17	0.112	0.530	0.448	No
ng4	4	Yes	3389.76	3483.59	0.124	0.646	0.443	No

Minimum class-specific average posterior probability refers to the lowest class-specific mean assigned posterior probability within each model. Relative entropy was used to summarize overall classification separation, with higher values indicating better classification quality. Model eligibility was defined as successful convergence, each class accounting for ≥5% of the sample, and a minimum class-specific class-specific average posterior probability >0.70. Detailed posterior classification quality is provided in Supplementary Table 2.

LCMM, latent class mixed model; TG, triglyceride; AIC, Akaike information criterion; BIC, Bayesian information criterion.

**Figure 1:**
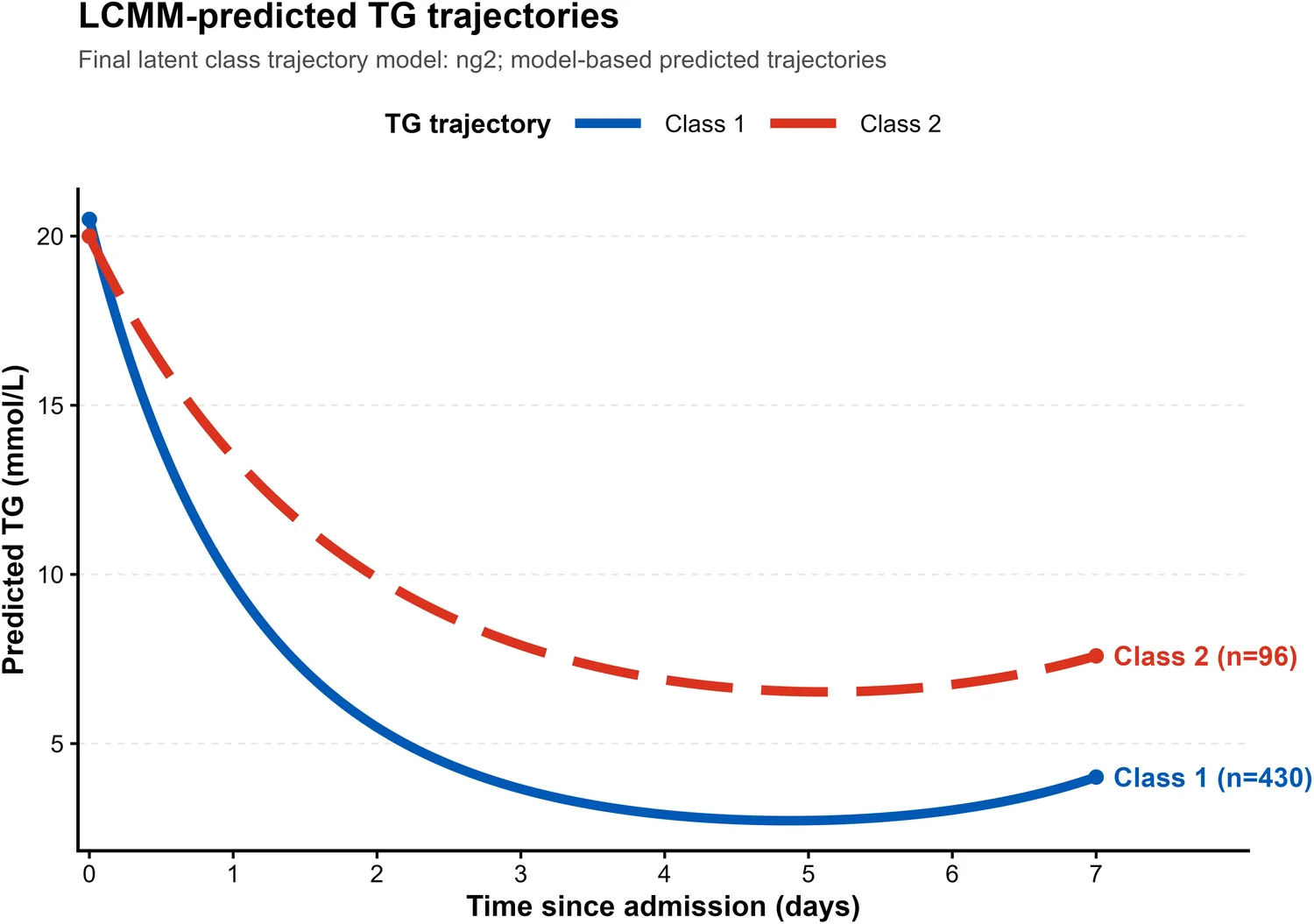
Dynamic TG trajectories during the first 7 days after admission were identified using a latent class mixed model (LCMM). Class 1 represents the rapid-decline TG trajectory group, and Class 2 represents the slow-decline TG trajectory group. The curves indicate estimated TG trends in each trajectory group, and the shaded areas represent 95% confidence intervals. TG, triglyceride; LCMM, latent class mixed model; CI, confidence interval.

Based on the final two-class model, two early in-hospital TG clearance trajectories were identified: a rapid-decline trajectory (*n* = 430, 81.7%) and a slow-decline trajectory (*n* = 96, 18.3%) (Figure 1).

### 3.2 Baseline characteristics and clinical outcomes according to TG trajectory group

Baseline characteristics according to TG trajectory group are presented in Table 1. Overall, the median age was 37 years (IQR: 32–43 years), and 412 patients were male. The median baseline TG level was 20.09 mmol/L (IQR: 12.56–31.96 mmol/L), the median maximum TG was 20.80 mmol/L (IQR: 13.48–32.21 mmol/L), and the median TWM-TG was 8.64 mmol/L (IQR: 6.26–12.06 mmol/L). Compared with the rapid-decline trajectory group, the slow-decline trajectory group had a higher TWM-TG value [13.00 mmol/L (IQR: 9.90–19.60 mmol/L) vs 7.99 mmol/L (IQR: 5.97–10.80 mmol/L), *P* < 0.001]. Blood urea nitrogen (BUN) levels were also higher in the slow-decline trajectory group than in the rapid-decline trajectory group [5.31 (IQR: 4.27–6.55) mmol/L vs 4.53 (IQR: 3.70–5.70) mmol/L, *P* < 0.001], and the prevalence of diabetes was higher in the slow-decline trajectory group (64.6% vs 50.7%, *P* = 0.019). The maximum TG was numerically higher in the slow-decline trajectory group than in the rapid-decline trajectory group, although the between-group difference did not reach statistical significance [22.83 (IQR: 13.41–38.14) mmol/L vs 20.30 (IQR: 13.52–31.23) mmol/L, *P* = 0.071].

Regarding clinical outcomes, 96 patients (18.3%) in the study cohort had severe acute pancreatitis, and 149 patients (28.3%) had pancreatic necrosis. The proportion of patients with severe acute pancreatitis was significantly higher in the slow-decline trajectory group than in the rapid-decline trajectory group (46.9% vs 11.9%, *P* < 0.001). The proportion of patients with pancreatic necrosis was also higher in the slow-decline trajectory group than in the rapid-decline trajectory group (38.5% vs 26.0%, *P* = 0.020). Among the 149 patients diagnosed with pancreatic necrosis, all cases were confirmed by CECT, with a median confirmation time of 3 days after admission (IQR: 2–4 days; range: 1–13 days); 133 cases (89.3%) were confirmed within the first 7 days and 16 cases (10.7%) after day 7 (Supplementary Table 3).

### 3.3 Associations of TG trajectory and TWM-TG with severe acute pancreatitis and pancreatic necrosis

To construct multivariable-adjusted models, covariates for each outcome were selected using a structured variable-selection procedure. First, the Boruta algorithm was applied for initial variable selection. Variables confirmed by the Boruta algorithm were then entered into LASSO regression models with 10-fold cross-validation. Variables retained by LASSO regression were further assessed for multicollinearity, and no variable included in the final adjustment sets had a variance inflation factor (VIF) > 5.

For severe acute pancreatitis, the final adjustment variables included blood urea nitrogen, serum creatinine, serum calcium, procalcitonin, hematocrit, amylase, lipase, heart rate, and age. For pancreatic necrosis, the final adjustment variables included serum creatinine, high-density lipoprotein cholesterol, aspartate aminotransferase, blood urea nitrogen, serum calcium, procalcitonin, total protein, and hemoglobin (Supplementary Figures 1 and 2).

Firth logistic regression was used to analyze the associations between TG-related indicators and study outcomes. Models were fitted separately in the five imputed datasets, and effect estimates were pooled according to Rubin's rules. Because TG trajectory and TWM-TG were derived from TG measurements obtained during the first 7 days after admission, these analyses describe associations with clinical outcomes during the early in-hospital disease course rather than strictly pre-outcome prediction. The multivariable regression results for TG-related indicators and study outcomes are presented in Table 3. In the analysis of severe acute pancreatitis, a slow-decline TG trajectory remained significantly associated with higher odds of severe acute pancreatitis. Using the rapid-decline TG trajectory group as the reference, the slow-decline TG trajectory group had 6.44 times the odds of severe acute pancreatitis after multivariable adjustment (OR=6.44, 95% CI: 3.55–11.69, *P* < 0.001).

**Table 3:** Multivariable firth logistic regression analysis of TG-related indicators and study outcomes.

Outcome	TG-related indicator	Adjusted OR (95% CI)	P value	N
Pancreatic necrosis	TG trajectory: slow vs rapid decline	1.55 (0.94-2.54)	0.086	526
	TWM-TG, per SD	1.27 (1.05-1.54)	0.016	526
	TG_MAX, per SD	1.19 (0.98-1.44)	0.078	526
Severe AP	TG trajectory: slow vs rapid decline	6.44 (3.55-11.69)	<0.001	526
	TWM-TG, per SD	1.66 (1.33-2.08)	<0.001	526
	TG_MAX, per SD	1.21 (0.96-1.53)	0.112	526

Results are presented as ORs (95% CIs). Multivariable Firth logistic regression models were fitted in five imputed datasets and pooled using Rubin's rules. TWM-TG and maximum TG were standardized, and ORs are presented per 1-SD increase. The rapid-decline trajectory group was used as the reference group for TG trajectory. Models were adjusted for outcome-specific covariates selected using Boruta, LASSO regression, and VIF assessment.

OR, odds ratio; CI, confidence interval; SD, standard deviation; TG, triglyceride; TWM-TG, time-weighted mean triglyceride exposure.

TWM-TG also remained significantly associated with severe acute pancreatitis. After multivariable adjustment, each 1-standard-deviation increase in TWM-TG was associated with 66% higher odds of severe acute pancreatitis (OR=1.66, 95% CI: 1.33–2.08, *P* < 0.001). In contrast, maximum TG was not significantly associated with severe acute pancreatitis (OR=1.21, 95% CI: 0.96–1.53, *P* = 0.112).

**Figure 2:**
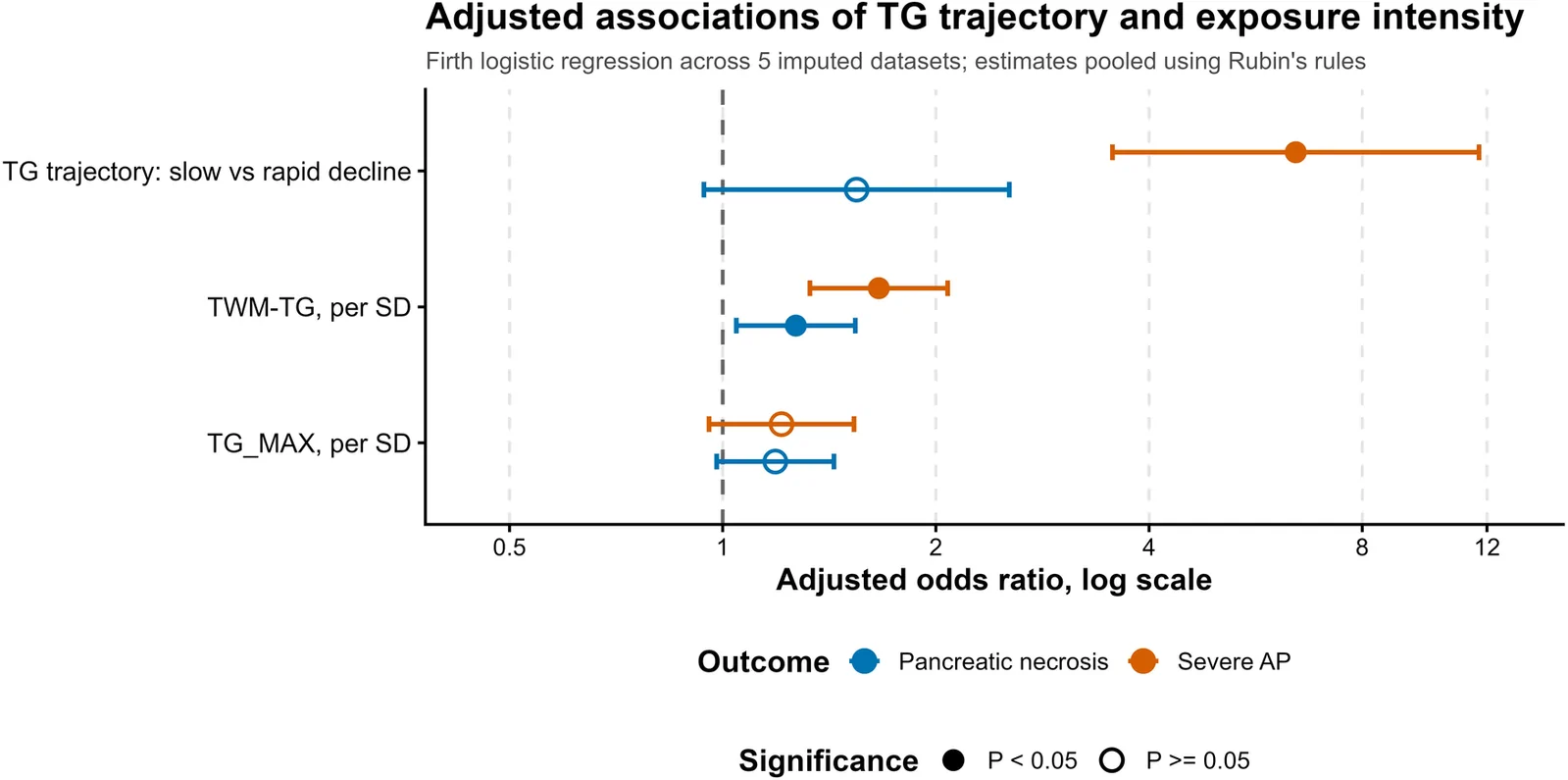
The forest plot shows the results of multivariable-adjusted Firth logistic regression. Effect estimates for TWM-TG and maximum TG were calculated per 1-standard-deviation increase; the rapid-decline trajectory group was used as the reference for TG trajectory. The horizontal axis represents ORs and 95% CIs. OR, odds ratio; CI, confidence interval; TG, triglyceride; TWMTG, time-weighted mean triglyceride exposure.

In the analysis of pancreatic necrosis, TWM-TG remained significantly associated with higher odds of pancreatic necrosis after multivariable adjustment (OR=1.27, 95% CI: 1.05–1.54, *P* = 0.016). Compared with the rapid-decline TG trajectory group, the slow-decline TG trajectory group did not show a statistically significant association with pancreatic necrosis (OR=1.55, 95% CI: 0.94–2.54, *P* = 0.086). The association between maximum TG and pancreatic necrosis also did not reach statistical significance (OR=1.19, 95% CI: 0.98–1.44, *P* = 0.078). A forest plot of the main regression results is shown in Figure 2.

### 3.4 Restricted cubic spline analysis of TWM-TG and clinical outcomes

**Figure 3:**
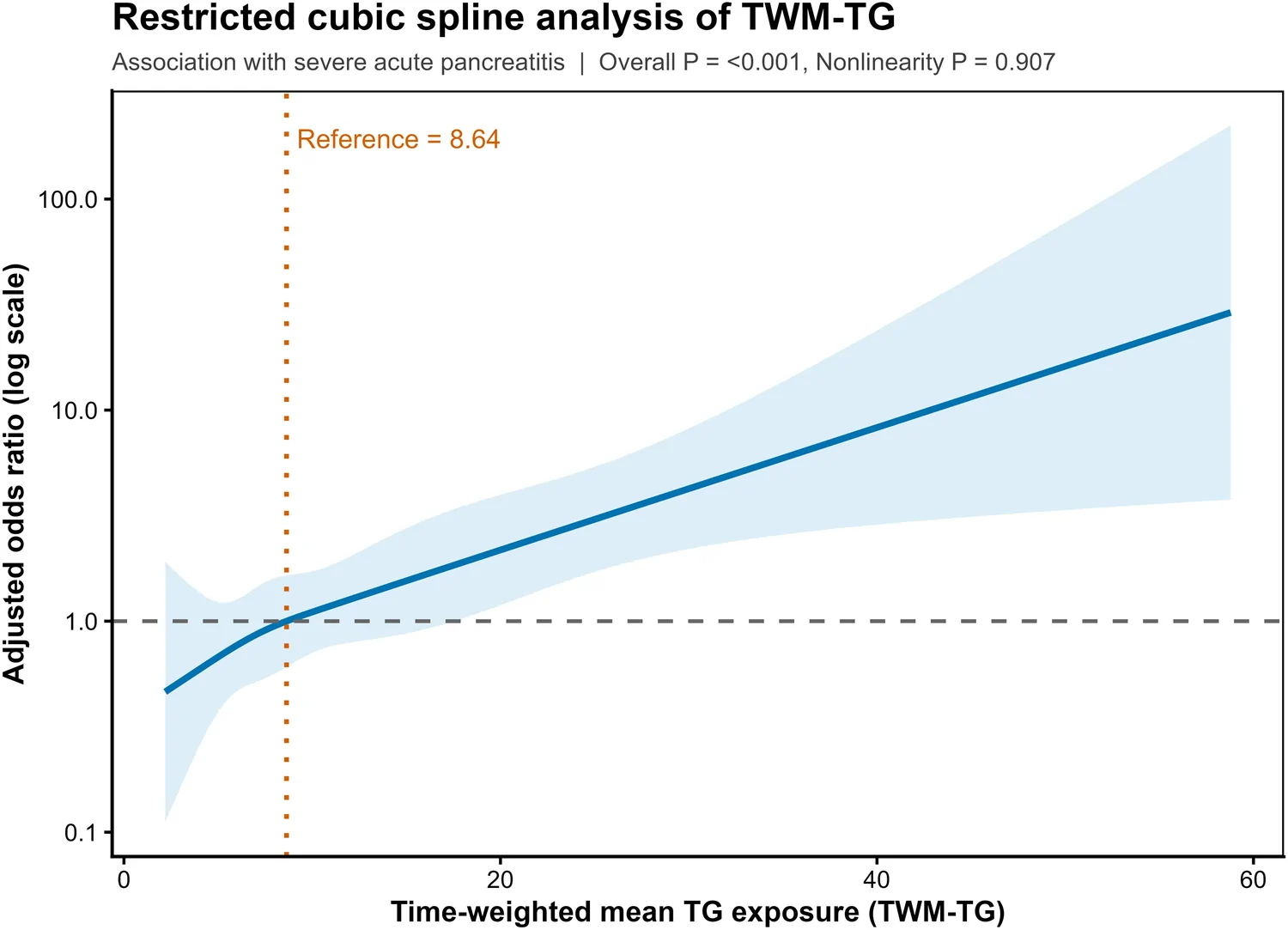
Restricted cubic spline analysis was used to assess the potential nonlinear association between TWM-TG and severe acute pancreatitis. The median TWM-TG value was used as the reference, and the model was adjusted for the same covariates as the multivariable model for severe acute pancreatitis. The shaded area represents the 95% confidence interval. Overall association *P* < 0.001; *P* for nonlinearity = 0.907.

Restricted cubic spline (RCS) analysis was performed to assess the potential nonlinear associations of TWM-TG with SAP and pancreatic necrosis. The RCS curve for the association between TWM-TG and SAP is shown in Figure 3. TWM-TG showed a significant overall association with SAP (overall *P* < 0.001), but no significant nonlinear association was observed (P for nonlinearity = 0.907). These findings suggest that, within the observed range in this study, the association between TWM-TG and SAP was approximately linear, without evidence of a clear threshold effect. Supplementary analyses showed that TWM-TG was also associated with pancreatic necrosis overall (overall *P* = 0.019), with no significant evidence of nonlinearity (P for nonlinearity = 0.105) (Supplementary Figure 3). These results support modeling TWM-TG as a linear continuous variable per 1-standard-deviation increase in the main regression models.

### 3.5 Exploratory ROC analysis with respect to severe acute pancreatitis

As a supplementary analysis, exploratory receiver operating characteristic (ROC) curve analysis was performed to compare the discriminatory information provided by static and dynamic TG-related indicators with respect to severe acute pancreatitis (SAP) (Supplementary Table 4 and Supplementary Figure 4). Across the five imputed datasets, the mean area under the curve (AUC) values for baseline TG and maximum TG when used alone were 0.519 (95% CI: 0.452–0.585) and 0.503 (95% CI: 0.436–0.570), respectively, indicating limited discrimination of these static TG indicators with respect to SAP. In contrast, TWM-TG and TG trajectory showed higher AUCs, with mean AUCs of 0.650 (95% CI: 0.587–0.713) and 0.675 (95% CI: 0.623–0.728), respectively. Exploratory DeLong tests performed in the first imputed dataset showed that the AUCs of TWM-TG (AUC difference = 0.131, *P* = 0.005) and TG trajectory (AUC difference = 0.157, *P* < 0.001) were significantly higher than that of baseline TG, whereas the difference between maximum TG and baseline TG was not statistically significant (*P* = 0.246).

The clinical covariate model had a mean AUC of 0.838 (95% CI: 0.787–0.889). After adding TWM-TG to the clinical covariate model, the mean AUC was 0.861 (95% CI: 0.814–0.908); after adding TG trajectory, the mean AUC was 0.877 (95% CI: 0.833–0.921). The combined model including clinical covariates, TG trajectory, and TWM-TG had the numerically highest mean AUC, at 0.881 (95% CI: 0.838–0.924). However, compared with the clinical covariate model alone, the increase in AUC for the combined model did not reach statistical significance in the DeLong test performed in the first imputed dataset (AUC difference = 0.043, *P* = 0.204). Taken together, these exploratory ROC results suggest that when evaluated individually, dynamic TG indicators showed higher AUCs than static TG measurements, whereas their incremental discriminatory value beyond clinical covariates was modest.

### 3.6 Subgroup analysis

**Figure 4:**
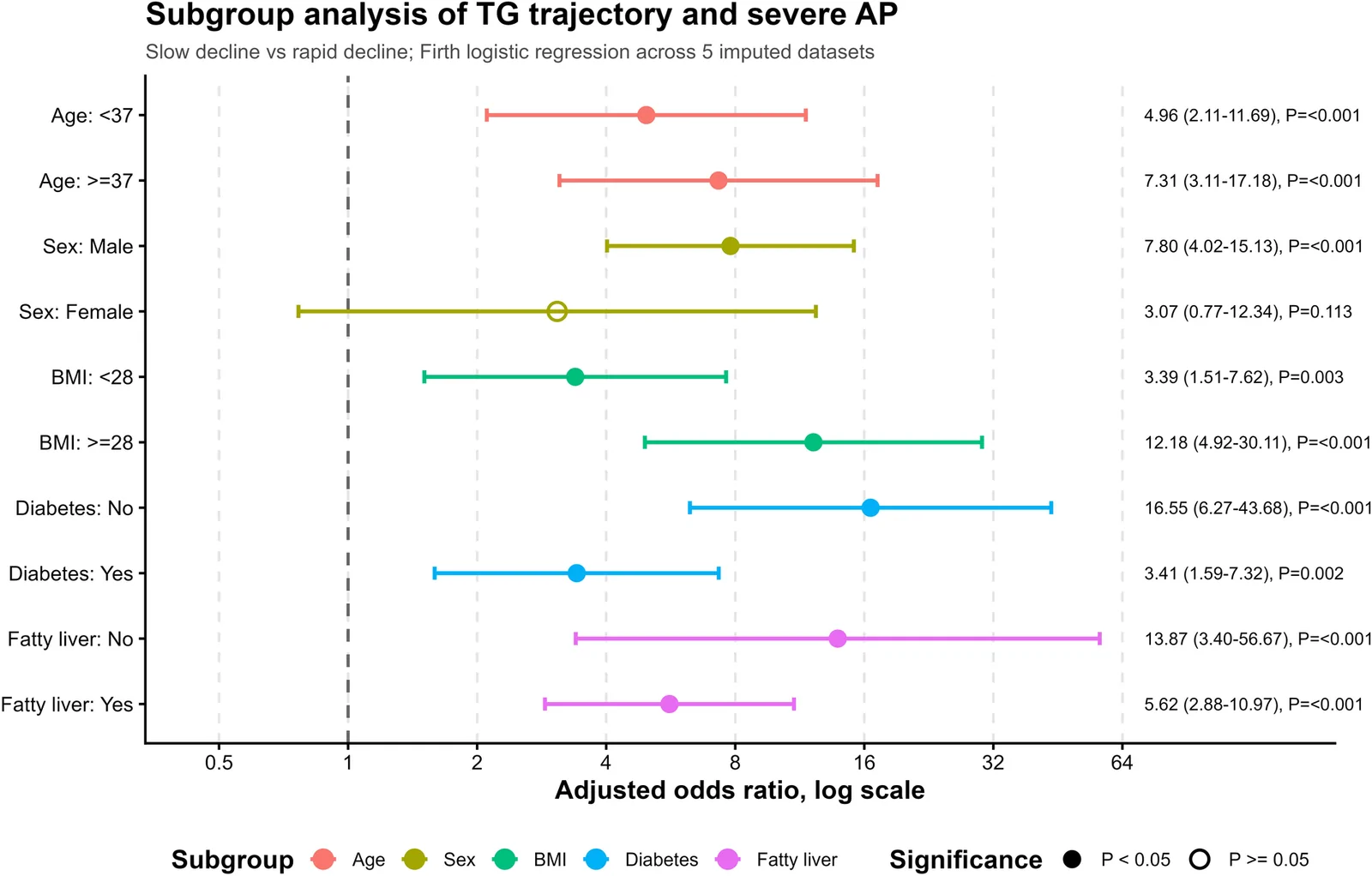
The rapid-decline TG trajectory group was used as the reference group. Points indicate adjusted odds ratios, and horizontal bars indicate 95% confidence intervals. Solid points indicate *P* < 0.05, and open points indicate *P* ≥ 0.05. Effect estimates were obtained from Firth logistic regression models fitted separately in the five imputed datasets and pooled using Rubin's rules. OR, odds ratio; CI, confidence interval; BMI, body mass index; TG, triglyceride; SAP, severe acute pancreatitis.

To evaluate whether the association between a slow-decline TG trajectory and severe acute pancreatitis (SAP) was consistent across patient subgroups defined by clinical characteristics, subgroup analyses were conducted according to age, sex, BMI, diabetes status, and fatty liver status (Figure 4 and Supplementary Table 5). Overall, the direction of the association was largely consistent across subgroups. Regardless of age (<37 years or ≥37 years) or fatty liver status, a slow-decline TG trajectory was significantly associated with higher odds of SAP. Regarding sex, this association was statistically significant among male patients; among female patients, the direction of the association was similar but did not reach statistical significance.

Diabetes status showed a significant interaction in this association (P for interaction = 0.005). Although higher odds of SAP were observed in both subgroups, the association with a slow-decline TG trajectory was stronger among patients without diabetes (OR=16.55, 95% CI: 6.27–43.68) than among those with diabetes (OR=3.41, 95% CI: 1.59–7.32). Regarding BMI, a slow-decline TG trajectory was associated with higher odds of SAP both among patients with BMI <28 kg/m^2^ (OR=3.39, 95% CI: 1.51–7.62) and those with BMI ≥28 kg/m^2^ (OR=12.18, 95% CI: 4.92–30.11), although the interaction did not reach statistical significance (P for interaction = 0.066). These subgroup findings should be considered exploratory and suggest that the association between a slow-decline TG trajectory and SAP was broadly consistent across clinically relevant strata, with possible heterogeneity according to diabetes status.

## 4 Discussion

This study systematically evaluated dynamic changes in TG levels during early hospitalization and their associations with disease severity and pancreatic necrosis in patients with hypertriglyceridemia-associated acute pancreatitis (HTG-AP). Using a latent class mixed model (LCMM), two broad TG trajectory patterns were identified: a rapid-decline trajectory and a slow-decline trajectory. A slow-decline TG trajectory remained significantly associated with higher odds of severe acute pancreatitis (SAP) after multivariable adjustment. TWM-TG was also associated with higher odds of SAP, whereas maximum TG showed only a nonsignificant association. For pancreatic necrosis, TWM-TG remained significantly associated with higher odds of pancreatic necrosis after multivariable adjustment, whereas the slow-decline TG trajectory and maximum TG did not reach statistical significance. Taken together, these findings suggest that early in-hospital TG clearance patterns and TWM-TG may capture different dimensions of the early in-hospital disease course in patients with HTG-AP.

The temporal pattern of TG clearance was another clinically relevant finding of this study. Although baseline or peak TG levels are commonly used to characterize lipid burden, they essentially reflect only a single time-point state within a dynamic metabolic process (8). In contrast, TG trajectory analysis can characterize both the direction and rate of change in lipid levels over time (29). In this study, the proportion of patients with SAP was higher in the slow-decline TG trajectory group than in the rapid-decline trajectory group. This association remained significant after adjustment for selected clinical covariates and after pooling estimates across multiple imputed datasets. Delayed TG clearance may therefore reflect or accompany persistent metabolic stress, sustained lipotoxic burden, metabolic-inflammatory disturbance, and early response to clinical management during the early phase of HTG-AP (10, 30). In addition, TG clearance during hospitalization may also be influenced by fasting, insulin therapy, lipid-lowering treatment, plasma exchange, nutritional strategy, fluid resuscitation, and ICU-level care. Thus, the LCMM classification should be viewed as an empirical summary of early TG clearance patterns, rather than evidence of discrete pathophysiological subtypes.

In addition to the TG clearance trajectory, TWM-TG represents the time-normalized average intensity of TG exposure during the actual observation period. Compared with crude cumulative exposure, TWM-TG is standardized by the time interval between the first and last TG measurements, which may partially reduce the influence of interpatient differences in the actual observation time span on exposure estimation. In this study, after multivariable adjustment, each 1-standard-deviation increase in TWM-TG was associated with higher odds of SAP, whereas maximum TG was not significantly associated with SAP. This finding suggests that a persistently higher average TG burden may capture information not reflected by a single peak value (3). Broader lipid-metabolism studies also underscore the biological relevance of lipid dysregulation in hyperlipidemia and related metabolic conditions (31, 32). From a biological perspective, sustained TG burden may be consistent with free fatty acid–mediated lipotoxicity, pancreatic microcirculatory impairment, and amplification of systemic inflammatory responses; however, in the context of this retrospective study, TWM-TG should be interpreted as a dynamic marker of the early metabolic-inflammatory disease course rather than as a confirmed causal determinant of organ dysfunction (13).

Regarding discriminatory performance, exploratory ROC analysis showed that baseline TG or maximum TG alone had limited ability to discriminate severe acute pancreatitis (SAP). In contrast, the mean AUCs for TWM-TG and TG trajectory were 0.650 and 0.675, respectively, and exploratory DeLong testing showed that both AUCs were significantly higher than the AUC for baseline TG. These findings indicate that TG exposure intensity and clearance patterns may complement static TG measurements. After TG trajectory and TWM-TG were added to the clinical covariate model, the model AUC increased from 0.838 to 0.881; however, this improvement did not reach statistical significance in the DeLong test. Therefore, early dynamic TG-related indicators may provide supplementary information for clinical assessment of disease severity, but they should not be interpreted as standalone prediction tools or substitutes for comprehensive clinical judgment.

Restricted cubic spline analysis showed a significant overall association between TWM-TG and SAP, without significant evidence of nonlinearity. This suggests that, within the observed range in this study, the association was approximately linear, without evidence of a clear threshold effect, supporting the modeling of TWM-TG as a linear continuous variable in the main analysis. A similar pattern was observed for pancreatic necrosis, with an overall association but no statistically significant evidence of nonlinearity.

Compared with SAP, the association patterns between TG-related indicators and pancreatic necrosis differed to some extent. After multivariable adjustment, only TWM-TG remained significantly associated with higher odds of pancreatic necrosis, whereas the slow-decline TG trajectory and maximum TG did not reach statistical significance. This finding suggests that TWM-TG may capture aspects of local pancreatic injury that are not fully represented by the clearance trajectory or peak TG alone. One possible explanation is that pancreatic necrosis is influenced not only by systemic inflammatory responses and metabolic disturbances but also by local microcirculatory impairment, insufficient tissue perfusion, and free fatty acid–mediated local toxic injury (14). Therefore, the association between TWM-TG and pancreatic necrosis may indicate that persistently higher TG burden during the early in-hospital disease course reflects or accompanies more pronounced local pancreatic injury, although further validation in external cohorts is needed.

In further analyses of the primary outcome, the association between a slow-decline TG trajectory and SAP showed a generally consistent direction across subgroups defined by age, sex, BMI, diabetes status, and fatty liver status. Diabetes status showed a significant interaction, with a stronger association observed in patients without diabetes than in those with diabetes. The reasons for this heterogeneity remain uncertain. Differences in baseline metabolic status or early in-hospital management between patients with and without diabetes may have contributed to the observed difference (33). Given the high prevalence of diabetes in this cohort, diabetes-related treatment background may also be relevant when interpreting TG clearance. Recent literature has drawn attention to the pancreatic safety of GLP-1 receptor agonists, although these agents were not specifically evaluated in the present study (34). However, this subgroup finding remains exploratory and hypothesis-generating and requires further validation in external cohorts (35).

This study has several strengths. First, TG dynamics were characterized using serial measurements rather than single baseline or peak TG values. TWM-TG was calculated to quantify time-normalized average TG exposure intensity during the actual observation period, and LCMM was used to identify early in-hospital TG clearance trajectories, thereby capturing both exposure intensity and clearance pattern. Second, covariates were selected using a structured procedure combining the Boruta algorithm, LASSO regression, and VIF assessment, and the main regression models were fitted using Firth logistic regression with multiple imputation. The structured variable-selection procedure improved the transparency and reproducibility of model construction, while Firth regression and multiple imputation were used to address potential small-sample estimation bias and missing data, respectively. Finally, exploratory ROC analysis suggested that dynamic TG-related indicators may complement static TG measurements in the assessment of disease severity.

This study has several limitations. First, temporal ambiguity between TG-related indicators and clinical outcomes should be considered. TG trajectories and TWM-TG were constructed using TG measurements obtained during the first 7 days after admission, whereas SAP and pancreatic necrosis may develop or be clinically recognized during the same period. Therefore, delayed TG clearance and elevated TWM-TG may partly reflect evolving disease severity, treatment response, or complications already underway rather than strictly pre-outcome predictors. Reverse causation and post-outcome exposure bias cannot be completely excluded, and these TG-related indicators should be interpreted as dynamic markers of the early in-hospital disease course rather than causal determinants of severe complications.

Second, this was a retrospective single-center cohort study, and selection bias may have been present. In particular, the exclusion of patients with a hospital stay of <7 days may have led to underrepresentation of patients with early death, early transfer, rapid recovery and early discharge, or insufficient longitudinal TG monitoring. Therefore, the findings are most applicable to patients hospitalized long enough to undergo repeated TG assessment during the first week after admission.

Third, TG measurements were obtained during routine clinical practice rather than according to a fixed sampling protocol, and the timing and frequency of testing were not fully consistent across patients. Although TG measurement time points and observation intervals were generally comparable between the rapid-decline and slow-decline trajectory groups, informative monitoring related to disease severity may still have influenced LCMM classification and TWM-TG estimation. TWM-TG was used to partially reduce the influence of differences in the actual observation time span on TG burden estimation, but it cannot completely eliminate the influence of nonuniform sampling intervals.

Fourth, although missing data were handled using MICE and covariates were selected using the Boruta algorithm, LASSO regression, and VIF assessment, the variable-selection procedure remained data-driven, and residual confounding or overfitting cannot be fully excluded. In addition, some adjusted covariates, such as renal function, serum calcium, inflammatory markers, hematocrit, and heart rate, may also reflect early disease severity. Therefore, the adjusted associations should not be interpreted as causal effect estimates. Unmeasured or incompletely captured clinical factors may still exist, including differences in fluid resuscitation strategies, insulin therapy, lipid-lowering medication use, timing of plasma exchange, nutritional support, ICU care, and other treatment intensities.

Fifth, pancreatic necrosis was a secondary outcome, and its assessment may have been influenced by the timing, clinical indications, and frequency of CECT examinations. Because CECT was not performed according to a uniform study imaging protocol, indication-related imaging bias and differences in the timing of necrosis confirmation cannot be fully excluded. Future studies may further integrate quantitative imaging features and dynamic changes in inflammatory markers.

Sixth, the ROC analysis in this study was exploratory, and the DeLong test was performed only in the first imputed dataset. Therefore, the incremental discriminatory value of early dynamic TG-related indicators over the clinical covariate model should be interpreted with caution and should not be considered validation of a clinical prediction model. External validation, calibration assessment, and decision-curve analysis are needed before these indicators can be incorporated into formal clinical prediction frameworks.

Finally, this was a single-center cohort from China, and external validity may be influenced by differences in patient characteristics, treatment protocols, ICU admission practices, and plasma exchange thresholds across institutions. The TG trajectory patterns identified in this study, the findings related to TWM-TG, and their clinical relevance as dynamic disease-course markers require further validation in large, multicenter external cohorts.

## 5 Conclusion

In summary, the early dynamic evolution of TG levels during hospitalization may provide clinically relevant information for assessing the disease course in patients with HTG-AP. Delayed TG clearance and elevated TWM-TG were both associated with higher odds of SAP after multivariable adjustment. In addition, TWM-TG was associated with higher odds of pancreatic necrosis, whereas maximum TG showed no significant association. TG clearance trajectories and TWM-TG may complement isolated baseline or peak TG measurements in characterizing sustained metabolic burden. Further multicenter validation is needed to confirm the generalizability and clinical relevance of these findings.

## Data Availability

The original contributions presented in the study are included in the article/Supplementary material. Further inquiries can be directed to the corresponding author.
