# Supplementary material for: Early in-hospital triglyceride clearance trajectories and time-weighted mean exposure in hypertriglyceridemia-associated acute pancreatitis: associations with disease severity and pancreatic necrosis
Source: Front Med (Lausanne). 2026 Sep 9;13:1921788. doi: 10.3389/fmed.2026.1921788 (PMC13597899; doi:10.3389/fmed.2026.1921788)
Supplement: Supplementary file 1 [file Table1.docx]

**Supplementary Figure 1.** Covariate selection process for severe acute pancreatitis

A, Boruta random forest feature selection for severe acute pancreatitis; B, 10-fold cross-validated LASSO regression based on Boruta-confirmed variables; C, LASSO coefficient path plot. Variables retained by LASSO were further assessed for multicollinearity using variance inflation factors. SAP, severe acute pancreatitis; LASSO, least absolute shrinkage and selection operator; VIF, variance inflation factor.

**Supplementary Figure 2.** Covariate selection process for pancreatic necrosis

A, Boruta random forest feature selection for pancreatic necrosis; B, 10-fold cross-validated LASSO regression based on Boruta-confirmed variables; C, LASSO coefficient path plot. Variables retained by LASSO were further assessed for multicollinearity using variance inflation factors. LASSO, least absolute shrinkage and selection operator; VIF, variance inflation factor.

**Supplementary Figure 3.** Restricted Cubic Spline Analysis of TWM-TG and Pancreatic Necrosis

The curve shows the association between TWM-TG and pancreatic necrosis estimated using restricted cubic spline analysis. The median TWM-TG value was used as the reference, and the model was adjusted using the covariates included in the multivariable model for pancreatic necrosis. The shaded area represents the 95% confidence interval. The overall association was statistically significant $\text{P}\text{=0.019}$, whereas no significant evidence of nonlinearity was observed (P for nonlinearity = 0.105). TWM-TG, time-weighted mean triglyceride exposure; CI, confidence interval.

**Supplementary Table 1**. Distribution of TG measurement time points used for trajectory modeling during the first 7 days after admission

| **Stratification** | **Group** | **n** | **No. of TG measurement time points, median (IQR)** | **First TG measurement time, days, median (IQR)** | **Last TG measurement time, days, median (IQR)** | **Observation interval, days, median (IQR)** |
| --- | --- | --- | --- | --- | --- | --- |
| TG trajectory | Rapid-decline trajectory | 430 | 3 (3–3) | 0.05 (0.03–0.07) | 5.50 (3.73–6.43) | 5.43 (3.70–6.33) |
| TG trajectory | Slow-decline trajectory | 96 | 3 (3–4) | 0.05 (0.03–0.08) | 5.62 (4.54–6.32) | 5.54 (4.48–6.24) |
| Severe acute pancreatitis | Non-severe | 430 | 3 (3–3) | 0.05 (0.03–0.08) | 5.62 (4.42–6.46) | 5.55 (4.28–6.35) |
| Severe acute pancreatitis | Severe | 96 | 4 (3–4) | 0.05 (0.03–0.08) | 4.00 (2.75–5.79) | 3.92 (2.70–5.70) |
| Pancreatic necrosis | Necrosis | 149 | 3 (3–4) | 0.05 (0.03–0.07) | 5.39 (3.35–6.43) | 5.15 (3.26–6.33) |
| Pancreatic necrosis | No necrosis | 377 | 3 (3–3) | 0.05 (0.03–0.08) | 5.55 (4.26–6.39) | 5.50 (4.10–6.29) |

TG, triglyceride; IQR, interquartile range. TG measurement time points refer to the TG records used for trajectory modeling during the first 7 days after admission.

**Supplementary Table 2.** Posterior Classification Quality of Candidate LCMMs With 2, 3, and 4 Classes

**Panel A. Class-specific distribution of assigned posterior probabilities**

| **Model** | **Assigned trajectory class** | **n** | **%** | **Mean assigned posterior probability** | **Median assigned posterior probability (IQR)** | **Minimum assigned posterior probability** | **Relative entropy** |
| --- | --- | --- | --- | --- | --- | --- | --- |
| ng2 | Class 1 / Rapid-decline trajectory | 430 | 81.7 | 0.877 | 0.929 (0.812–0.980) | 0.503 | 0.533 |
| ng2 | Class 2 / Slow-decline trajectory | 96 | 18.3 | 0.784 | 0.816 (0.639–0.929) | 0.502 | 0.533 |
| ng3 | Class 1 | 102 | 19.4 | 0.530 | 0.522 (0.480–0.576) | 0.386 | 0.448 |
| ng3 | Class 2 | 365 | 69.4 | 0.785 | 0.815 (0.664–0.917) | 0.348 | 0.448 |
| ng3 | Class 3 | 59 | 11.2 | 0.782 | 0.826 (0.655–0.943) | 0.443 | 0.448 |
| ng4 | Class 1 | 303 | 57.6 | 0.646 | 0.667 (0.564–0.741) | 0.367 | 0.443 |
| ng4 | Class 2 | 84 | 16.0 | 0.724 | 0.734 (0.615–0.871) | 0.423 | 0.443 |
| ng4 | Class 3 | 65 | 12.4 | 0.708 | 0.705 (0.582–0.854) | 0.345 | 0.443 |
| ng4 | Class 4 | 74 | 14.1 | 0.778 | 0.804 (0.629–0.937) | 0.452 | 0.443 |

**Panel B. Posterior probability matrix for candidate LCMMs**

| **Model** | **Assigned trajectory class** | **n** | **Mean posterior probability for Class 1** | **Mean posterior probability for Class 2** | **Mean posterior probability for Class 3** | **Mean posterior probability for Class 4** |
| --- | --- | --- | --- | --- | --- | --- |
| ng2 | Class 1 / Rapid-decline trajectory | 430 | 0.877 | 0.123 | — | — |
| ng2 | Class 2 / Slow-decline trajectory | 96 | 0.216 | 0.784 | — | — |
| ng3 | Class 1 | 102 | 0.530 | 0.261 | 0.209 | — |
| ng3 | Class 2 | 365 | 0.165 | 0.785 | 0.050 | — |
| ng3 | Class 3 | 59 | 0.108 | 0.111 | 0.782 | — |
| ng4 | Class 1 | 303 | 0.646 | 0.083 | 0.176 | 0.095 |
| ng4 | Class 2 | 84 | 0.165 | 0.724 | 0.076 | 0.034 |
| ng4 | Class 3 | 65 | 0.185 | 0.007 | 0.708 | 0.101 |
| ng4 | Class 4 | 74 | 0.140 | 0.010 | 0.073 | 0.778 |

LCMM, latent class mixed model; IQR, interquartile range. Assigned posterior probability refers to the posterior probability corresponding to the class to which each patient was assigned. Relative entropy was calculated at the model level and is therefore identical across classes within the same model. The ng2 model was selected as the final trajectory model. For the ng2 model, Class 1 and Class 2 corresponded to the rapid-decline and slow-decline TG trajectory groups, respectively. The ng3 and ng4 models were candidate models and were not retained as the final trajectory model.

**Supplementary Table 3**. Timing of contrast-enhanced CT confirmation among patients with pancreatic necrosis

| **Variable** | **Pancreatic necrosis patients** |
| --- | --- |
| Number of patients with pancreatic necrosis | 149 |
| Imaging modality used for necrosis confirmation | Contrast-enhanced CT, 149/149 |
| Time from admission to necrosis-confirming CECT, median (IQR), days | 3 (2–4) |
| Range, days | 1–13 |
| Necrosis confirmed ≤7 days after admission | 133/149 (89.3%) |
| Necrosis confirmed >7 days after admission | 16/149 (10.7%) |

CECT, contrast-enhanced computed tomography; IQR, interquartile range.

**Supplementary Table 4**. Exploratory ROC analysis for severe acute pancreatitis

| **Model** | **Mean AUC** | **95% CI** | **AUC range across imputed datasets** | **Mean sample size** | **Mean events** |
| --- | --- | --- | --- | --- | --- |
| Baseline TG | 0.519 | 0.452–0.585 | 0.519–0.519 | 526 | 96 |
| Maximum TG | 0.503 | 0.436–0.570 | 0.503–0.503 | 526 | 96 |
| TWM-TG | 0.650 | 0.587–0.713 | 0.650–0.650 | 526 | 96 |
| TG trajectory | 0.675 | 0.623–0.728 | 0.675–0.675 | 526 | 96 |
| Clinical covariates | 0.838 | 0.787–0.889 | 0.836–0.839 | 526 | 96 |
| Clinical covariates + TWM-TG | 0.861 | 0.814–0.908 | 0.860–0.862 | 526 | 96 |
| Clinical covariates + TG trajectory | 0.877 | 0.833–0.921 | 0.875–0.878 | 526 | 96 |
| Clinical covariates + TG trajectory + TWM-TG | 0.881 | 0.838–0.924 | 0.880–0.882 | 526 | 96 |

AUCs were calculated separately in the five imputed datasets and summarized as mean AUC, mean 95% CI, and AUC range. DeLong tests were performed in the first imputed dataset for exploratory comparisons of selected ROC curves. AUC, area under the curve; CI, confidence interval; TWM-TG, time-weighted mean triglyceride exposure; SAP, severe acute pancreatitis.

**Supplementary Figure 4.** ROC Curves of TG-Related Indicators and Clinical Models With Respect to Severe Acute Pancreatitis

ROC curves were plotted using the first imputed dataset as an exploratory analysis. Single TG-related indicators, including baseline TG, maximum TG, TWM-TG, and TG trajectory, were evaluated directly using their corresponding values or group assignments. Clinical covariate models and combined models were evaluated using individual model-derived fitted probabilities from Firth logistic regression models. ROC, receiver operating characteristic; TG, triglyceride; TWM-TG, time-weighted mean triglyceride exposure; AUC, area under the curve; SAP, severe acute pancreatitis.

**Supplementary Table 5.** Subgroup Analysis of the Association Between Slow-Decline TG Trajectory and Severe Acute Pancreatitis

| **Subgroup** | **Level** | **Mean sample size** | **Mean events** | **OR (95% CI)** | **P value** | **P for interaction** |
| --- | --- | --- | --- | --- | --- | --- |
| Age | <37 years | 250.8 | 54 | 4.96 (2.11–11.69) | <0.001 | 0.373 |
|  | ≥37 years | 275.2 | 42 | 7.31 (3.11–17.18) | <0.001 |  |
| Sex | Male | 412 | 74 | 7.80 (4.02–15.13) | <0.001 | 0.138 |
|  | Female | 114 | 22 | 3.07 (0.77–12.34) | 0.113 |  |
| BMI | <28 kg/m² | 279.6 | 46 | 3.39 (1.51–7.62) | 0.003 | 0.066 |
|  | ≥28 kg/m² | 246.4 | 50 | 12.18 (4.92–30.11) | <0.001 |  |
| Diabetes | No | 246 | 36 | 16.55 (6.27–43.68) | <0.001 | 0.005 |
|  | Yes | 280 | 60 | 3.41 (1.59–7.32) | 0.002 |  |
| Fatty liver | No | 107 | 28 | 13.87 (3.40–56.67) | <0.001 | 0.172 |
|  | Yes | 419 | 68 | 5.62 (2.88–10.97) | <0.001 |  |

BMI, body mass index; CI, confidence interval; OR, odds ratio; SAP, severe acute pancreatitis; TG, triglyceride. ORs represent the association between a slow-decline TG trajectory and severe acute pancreatitis, using the rapid-decline TG trajectory as the reference group. Subgroup analyses were performed in five imputed datasets, and estimates were pooled according to Rubin’s rules. Mean sample size and mean events are summarized across the imputed datasets. P for interaction was obtained from interaction terms between TG trajectory and the corresponding subgroup variable.
